# Genetic architecture and key regulatory genes of fatty acid composition in Gushi chicken breast muscle determined by GWAS and WGCNA

**DOI:** 10.1186/s12864-023-09503-1

**Published:** 2023-08-03

**Authors:** Shengxin Fan, Pengtao Yuan, Shuaihao Li, Hongtai Li, Bin Zhai, Yuanfang Li, Hongyuan Zhang, Jinxin Gu, Hong Li, Yadong Tian, Xiangtao Kang, Yanhua Zhang, Guoxi Li

**Affiliations:** 1 College of Animal Science and Technology, Henan Agricultural University, No.15 Longzihu University Area, Zhengzhou New District, Zhengzhou, 450002 China; 2 School of Medicine and Health, Harbin Institute of Technology, Harbin, 150001 HeiLongJiang China; 3 Zhengzhou Research Institute, Harbin Institute of Technology, Zhengzhou, 450000 Henan China; 4 Henan Key Laboratory for Innovation and Utilization of Chicken Germplasm Resources, Henan Agricultural University, No.15 Longzihu University Area, Zhengzhou New District, Zhengzhou, 450002 China; 5 The Shennong Laboratory, Zhengzhou, 450002 China

**Keywords:** Chicken, Breast muscle, Fatty acid composition, Genetic structure, GWAS, WGCNA

## Abstract

**Background:**

Fatty acids composition in poultry muscle is directly related to its tenderness, flavour, and juiciness, whereas its genetic mechanisms have not been elucidated. In this study, the genetic structure and key regulatory genes of the breast muscle fatty acid composition of local Chinese chicken, Gushi-Anka F2 resource population by integrating genome-wide association study (GWAS) and weighted gene co-expression network analysis (WGCNA) strategies. GWAS was performed based on 323,306 single nucleotide polymorphisms (SNPs) obtained by genotyping by sequencing (GBS) method and 721 chickens from the Gushi-Anka F2 resource population with highly variable fatty acid composition traits in the breast muscle. And then, according to the transcriptome data of the candidate genes that were obtained and phenotypic data of fatty acid composition traits in breast muscle of Gushi chickens at 14, 22, and 30 weeks of age, we conducted a WGCNA.

**Results:**

A total of 128 suggestive significantly associated SNPs for 11 fatty acid composition traits were identified and mapped on chromosomes (Chr) 2, 3, 4, 5, 13, 17, 21, and 27. Of these, the two most significant SNPs were Chr13:5,100,140 (*P* = 4.56423e-10) and Chr13:5,100,173 (*P* = 4.56423e-10), which explained 5.6% of the phenotypic variation in polyunsaturated fatty acids (PUFA). In addition, six fatty acid composition traits, including C20:1, C22:6, saturated fatty acid (SFA), unsaturated fatty acids (UFA), PUFA, and average chain length (ACL), were located in the same QTL intervals on Chr13. We obtained 505 genes by scanning the linkage disequilibrium (LD) regions of all significant SNPs and performed a WGCNA based on the transcriptome data of the above 505 genes. Combining two strategies, 9 hub genes (*ENO1*, *ADH1, ASAH1, ADH1C, PIK3CD, WISP1, AKT1*, *PANK3*, and *C1QTNF2*) were finally identified, which could be the potential candidate genes regulating fatty acid composition traits in chicken breast muscle.

**Conclusion:**

The results of this study deepen our understanding of the genetic mechanisms underlying the regulation of fatty acid composition traits, which is helpful in the design of breeding strategies for the subsequent improvement of fatty acid composition in poultry muscle.

**Supplementary Information:**

The online version contains supplementary material available at 10.1186/s12864-023-09503-1.

## Background

Muscle fatty acid composition is one of the most important meat-based food factors affecting meat quality and human health. Numerous human and animal studies have shown that PUFA can be transformed in vivo to produce a variety of derivatives that inhibit platelet agglutination [1, 2], increase platelet cell membrane fluidity, and change cell signaling, thereby inhibiting thrombosis formation [3, 4]. A high omega-6 fatty acid diet inhibits the anti-inflammatory and inflammatory mitigating effects of omega-3 fatty acids [5]. In general, the PUFA/SFA > 0.4, and the n-6/N-3 PUFA ratio maintained at (4–6)/1 is appropriate for human fat intake [6, 7]. Chicken is one of the most critical animal protein sources in people's dietary intake [8, 9]. The PUFA/SFA in chicken meat was higher than 0.4, whereas the n-6/n-3 was lower than 0.25, and thus, there was still a problem of fatty acid imbalance in the diet. Therefore, it is significant to reveal the genetic regulation mechanism of chicken muscle fatty acid composition and genetically improve chicken muscle fatty acid composition for improving meat quality and human health.

Muscle fatty acid composition traits are complex quantitative traits regulated by major and minor genes [10]. Candidate genes and regulatory loci for fatty acid composition traits in the muscle of livestock such as pigs, cattle, and sheep have been well studied [11–13]. These studies have shown that multiple genes control muscle fatty acid composition traits, and the regulatory loci were located on different chromosomes and regulated multiple fatty acid traits simultaneously. Compared with other animals and livestock, scientific studies lack the genetic regulation of fatty acid composition traits in chickens. Jin et al. identified 30 QTL related to fatty acid composition traits in leg and breast muscles [14]. However, only seven fatty acid-related QTLs, C18:0, C18:2, C16:0, C18:1, C18:3, C22:6, and C20:1, are currently recorded in the Chicken QTL Database (https://www.animalgenome.org/cgi-bin/QTLdb/GG/index). Muscle fatty acid deposition is essentially the esterification of fatty acids into triglycerides and deposition to body fat, involving the fine regulation of many aspects and genes related to fatty acid metabolism in the body [15]. Therefore, identifying these functional genes and their variant loci related to fatty acid metabolism is crucial to unraveling the genetic regulation of fatty acid composition traits in chickens.

The application of modern omics technology provides strong support for the genetic analysis of muscle fatty acid composition traits in livestock and poultry. GWAS has been widely used in the study of quantitative traits in livestock since Risch first proposed it in 1996 [16–18]. However, there is a lack of studies using chicken SNPs to systematically demonstrate associations between fatty acid traits in chicken and genomic loci. RNA sequencing (RNA-Seq) has been applied in the genetic resolution of fatty acid composition traits in livestock and poultry [19]. Yang et al. [20] screened several candidate genes affecting the percentage of PUFA in the thigh muscle of Huangshan black chickens by RNA-Seq, such as *FADS2*, *DCN*, *FRZB*, *OGN*, *PRKAG3*, *LHFP*, *CHCHD10*, *CYTL1*, *FBLN5,* and *ADGRD1*. Li et al. [21] identified 98 candidate genes regulating fatty acid composition in chicken breast muscle in six modules through WGCNA analysis. By WGCNA, two differentially expressed circRNAs and two competing endogenous RNAs can regulate chicken adipogenic differentiation [22]. These studies have deepened the understanding of the genetic regulation of fatty acid composition traits in chicken muscle. In recent years, multi-omics analysis strategies have been reported in the analysis of fatty acid composition traits in a few livestock and poultry, such as high-density SNP chip typing combined with phenotypic data to identify QTL, high-throughput SNP typing combined with RNA-seq data to identify expression quantitative trait loci (eQTL), phenotypic data and RNA-seq data association analysis to identify quantitative trait transcripts (QTTs) [23, 24]. These strategies have increased the depth and precision of fatty acid composition trait resolution, whereas there are fewer applications in chicken currently.

Gushi chicken is a local chicken breed for meat and eggs, with excellent characteristics such as tender meat and unique flavor [25]. To explore the excellent traits of Gushi Chicken, we constructed a Gushi-Anka F2 resource population, and a series of studies were previously carried out on intramuscular fat deposition in breast muscle of this breed from the aspects of the identification of key functional genes and regulation of non-coding RNA [21, 26, 27]. On this basis, the GBS sequencing data of 721 individuals from the F2 resource population, phenotypic data of 30 breast muscle fatty acid composition traits, and transcriptome profiles of breast muscle tissue of Gushi chicken*s* at 14, 22, and 30 weeks of age were used to analyze the genetic architecture and key regulatory genes of breast muscle fatty acid composition by integrating GWAS and WGCNA strategies. This study provides a valuable reference for a better understanding of the genetic regulation of fatty acid composition in breast muscle of Gushi chicken*s* and the molecular mechanisms underlying the formation of high-quality meat traits from the perspective of genetic variation and gene co-expression.

## Result

### Phenotype and genotype statistics

**Fig. 1:**
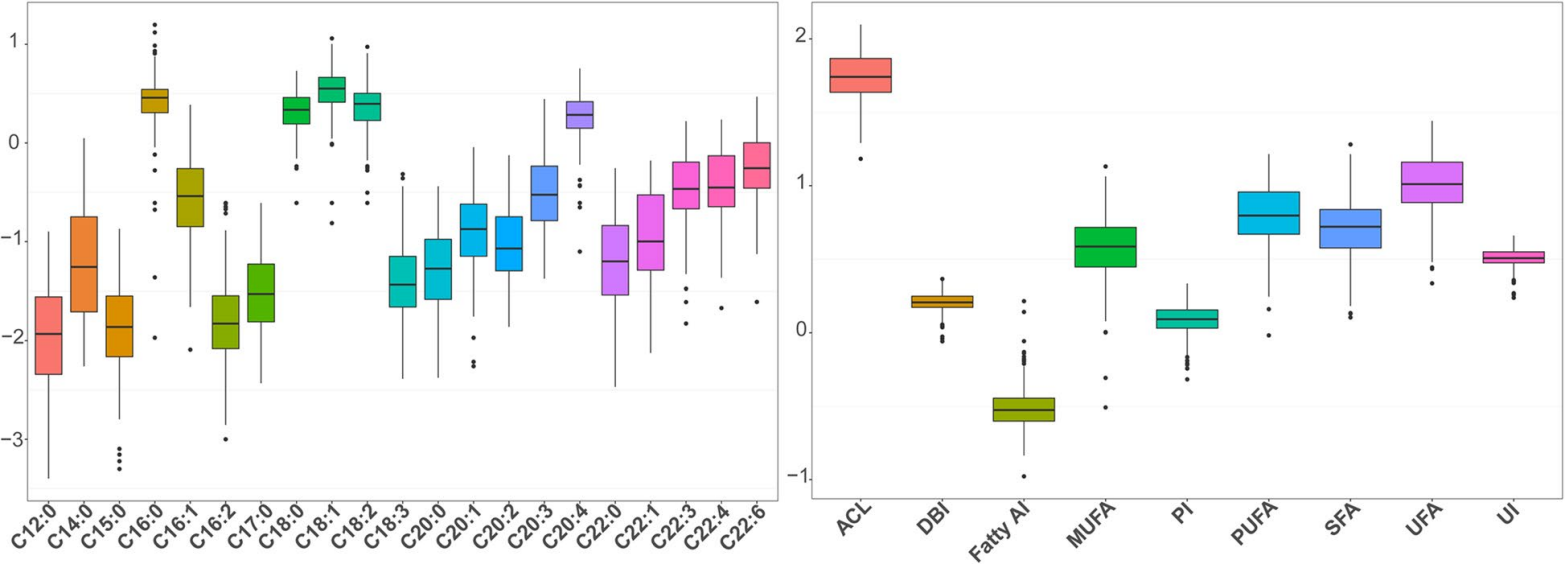
Box plots of the distribution of phenotypic values for fatty acid composition traits. The x-axis represents the fatty acid composition trait, and the y-axis represents the fatty acid content after log_10_ normalization

**Fig. 2:**
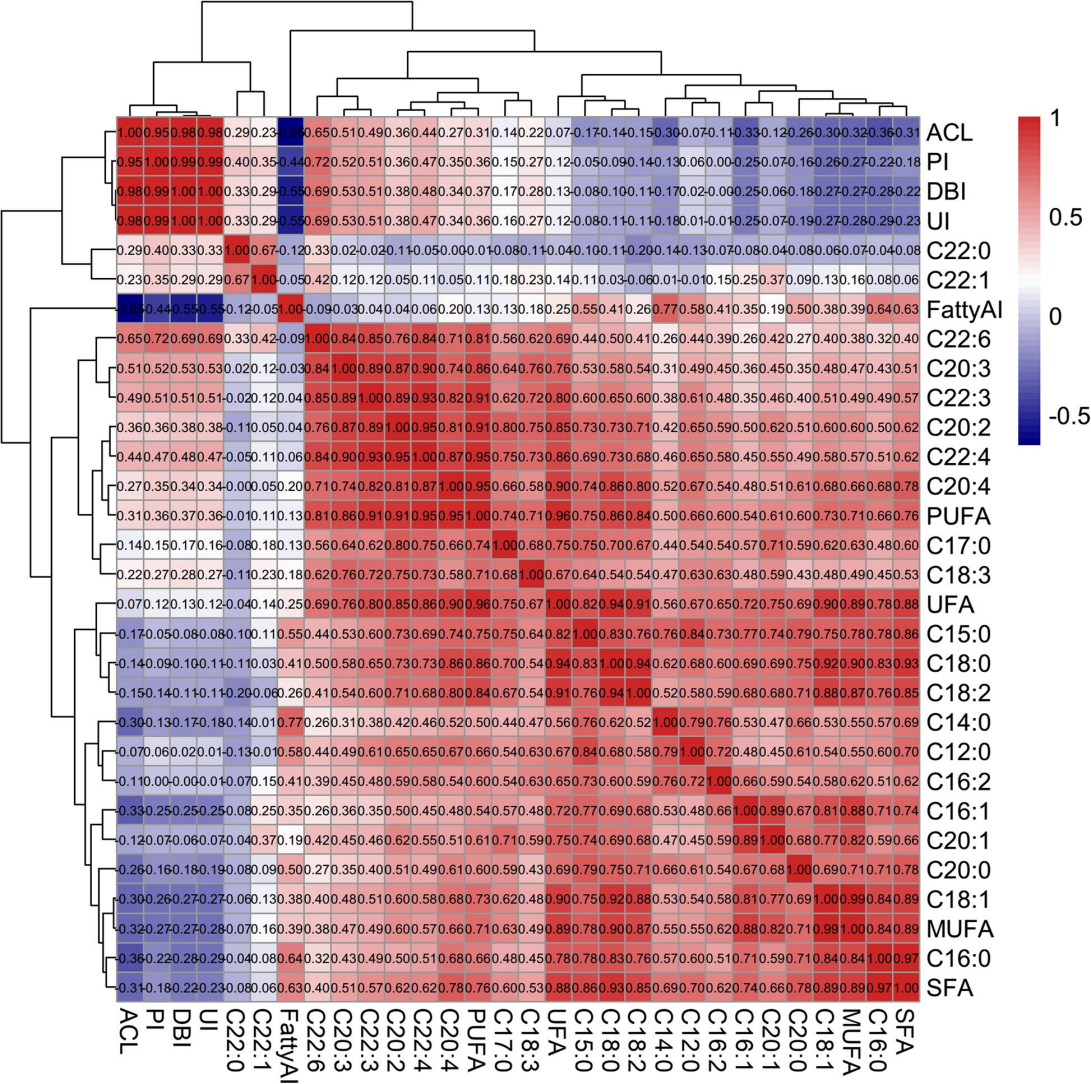
Pearson correlation between phenotypes. The Pearson correlation coefficients of 30 fatty acid traits were calculated, and the traits were clustered based on the correlation coefficients. The colors (numbers) represent the pairwise correlation coefficients of the fatty acid traits. Red indicates positive correlation, and blue indicates a negative correlation

Based on the contents of 21 fatty acids in breast muscle of the F2 resource population, 9 fatty acid metabolic traits were calculated, and phenotypic values of 30 fatty acid composition traits in breast muscle were obtained, including sample number, mean ± standard deviation, and heritability (h^2^) (Table 1). In the breast muscle tissue, the highest fatty acid content was C18:1, followed closely by C16:0, C18:2, and C18:0, accounting for approximately 66% of the total fatty acid content. The content of UFA was 2.16 times higher than the content of SFA. In addition, the contents of fatty acids in breast muscle of the F2 resource population showed significant population variation, especially C20:1, C22:0, C22:1, and other fatty acids could only be detected in part of individuals. This indicated that the fatty acid composition traits in breast muscle of the F2 resource population were separated (Fig. 1). The heritability of these 21 fatty acids was also estimated, with some fatty acid composition traits of moderate heritability (0.2–0.4), but most of them of low heritability (0–0.2), further suggesting that fatty acid composition traits in chicken muscle are complex quantitative traits that are regulated by a combination of micro-effective genes. Pearson correlations between fatty acid composition traits showed that all 21 fatty acid traits were correlated (Fig. 2), with those having the same number of carbon atoms being more correlated, in line with the principles of fatty acid carbon chain synthesis and elongation [28]. Otherwise, the four metabolic traits of ACL, peroxide index (PI), double bond index (DBI), and unsaturated index (UI), were highly correlated.

**Table 1:** Summary statistics for fatty acid compsition traits in breast muscle of the F2 resource population

Trait	N	Mean ± SD	h^2^	Trait	N	Mean ± SD	h^2^
Laurate C12:0	439	0.02 ± 0.04	0.034	Eicosadienoate C20:2	432	0.16 ± 0.35	0.049
Myristic C14:0	439	0.18 ± 0.33	0.08	Eicosatrienoate C20:3	430	0.55 ± 1.49	0.008
Pentadecanoate C15:0	439	0.02 ± 0.05	0.01	Arachidonate C20:4	439	2.14 ± 1.26	0.024
Palmitate C16:0	438	2.98 ± 1.58	0.25	Docosatrienoic C22:3	425	0.50 ± 0.49	0.023
Heptadecanoate C17:0	439	0.05 ± 0.10	0.01	Docosatetraenoate C22:4	429	0.52 ± 0.43	0.148
Stearate C18:0	439	2.30 ± 1.17	0.18	Docosahexaenoate C22:6	429	0.82 ± 0.72	0.01
Arachidate C20:0	391	0.09 ± 0.11	0.11	SFA content	438	5.51 ± 2.45	0.235
Behenate C22:0	350	0.12 ± 0.15	0.01	MUFA content	439	4.22 ± 2.06	0.16
Palmitoleate C16:1	439	0.52 ± 0.64	0.07	PUFA content	439	6.85 ± 2.92	0.198
Oleate C18:1	439	4.08 ± 2.79	0.001	UFA content	439	11.07 ± 4.66	0.205
Eicosenoate C20:1	157	0.22 ± 0.26	0.015	DBI	439	1.63 ± 0.23	0.167
Erucic acid C22:1	109	0.26 ± 0.38	0.01	ACL	439	18.27 ± 0.26	0.234
Palmitic C16:2	438	0.03 ± 0.09	0.028	UI	439	3.25 ± 0.47	0.167
Linoleate C18:2	439	2.77 ± 2.33	0.10	PI	439	1.26 ± 0.29	0.75
Linolenate C18:3	439	0.07 ± 0.15	0.001	Fatty AI	439	0.32 ± 0.13	0.01

N, The number of valid individuals for each fatty acid trait for analysis, h^2^ Heritability, *Mean* Arithmetic mean, *SD* Standard deviation

### GWAS for fatty acid traits

**Fig. 3:**
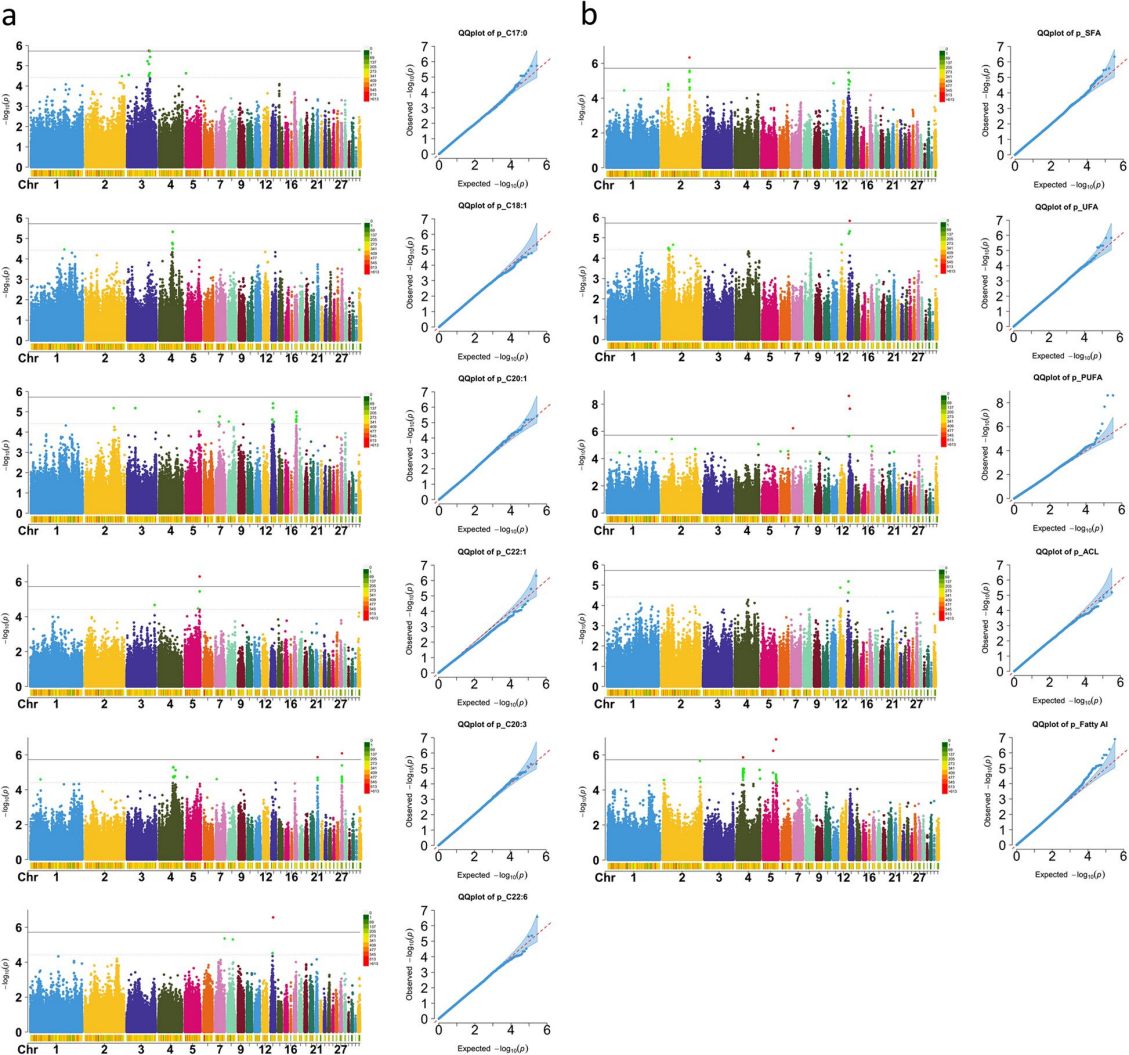
The Manhattan and Q-Q plots for eleven traits. **A** for six fatty acid composition traits, (**b**) for five fatty acid metabolic traits. Each dot in this figure corresponds to an SNP within the data set. In each Manhattan plot, the dot color indicates the chromosome on which the SNP is located, the dot position indicates the -log_10_-transformed *P* value of the SNP, the number below represents the chromosome number, the length of the figure above the number represents the length of the chromosome, and the color represents the number of SNPs on the chromosome. The solid and dashed lines represent genome-wide significance (-log_10_(*P*) > 5.72) threshold and chromosome-wide suggestive threshold (-log_10_(*P*) > 4.42), respectively. For each Q-Q plot, the *x-axis* represents the expected -log_10_-transformed *P* value, and the *y-axis* shows the observed -log_10_-transformed *P* value and the red line is the diagonal line

The imputed GWAS results of 30 fatty acid traits are shown in Fig. 3a, Fig. 3b, Table 2, Additional file 1:Fig. S1, Additional file 2:Fig. S2, and Additional file 5:Table S2. Annotation of each significant locus revealed putative candidate genes. Moreover, the genes located within the high-LD region (r^2^ > 0.3 and − log_10_(*P*) < 4.42) neighboring the significant locus also remained. The single marker analysis identified 16 SNPs above the genome-wide threshold and 128 above the chromosome-wide suggestive threshold. The 128 SNPs were associated with 11 fatty acid traits (C17:0, C18:1, C20:1, C22:1, C20:3, C22:6, SFA, UFA, PUFA, ACL, and fattyAI) and mapped on Chr2, 3, 4, 5, 13, 17, 21, and 27. Based on SNP annotation, we found 35 significant SNPs in the intergenic region, 64 in the introns, 4 upstream of the coding sequence, and 7 downstream. In the end, 505 candidate genes were screened within the linkage disequilibrium interval of these SNPs (Additional file 6:Table S3). In addition, an SNP was significantly associated with C20:1 located on the first exon of the gene *KCNT1* gene (Chr17: 8,537,914, Sorting Intolerant From Tolerant (SIFT) score = 0.03), which may lead to altering protein function.

**Table 2:** The significant SNPs and candidate genes associated with the fatty acid composition traits

**Chr**	**Trait**	**N**_**snp**_	**Top SNP**	*P*_**Value**	**Var (%)**	**Range (Mb)**	**Candidate gene**
2	C22:1	27	S2_24024322	4.83E-05	2	21.79–26.86	
	Fatty AI	5	S2_142073970	1.35E-06	1.7	14.14–14.40	*WISP1*
3	C17:0	12	S3_82848466	7.38E-07	1.2	82.56–86.52	
4	C18:1	6	S4_52115241	5.81E-05	1.7	50.57–52.25	
	C20:3	6	S4_55276199	1.28E-06	2	53.48–60.98	*ADH1C,ADH6, ELOVL6, FABP2, ASAH1*
5	Fatty AI	18	S5_50226872	1.90E-08	4.1	49.81–53.05	*AKT1*
	Fatty AI	6	S5_39758590	4.32E-08	4.1	38.64–39.86	
13	C20:1	4	S13_8349071	5.93E-05	1.3	41.37–41.59	
	C22:6	4	S13_10685870	3.41E-06	2	82.83–10.97	*C1QTNF2*
	SFA	6	S13_8144131	5.53E-06	1.6	50.37–89.19	*PANK3*
	UFA	12	S13_8121375	4.18E-07	1.7	51.00–88.70	*PANK3*
	PUFA	4	S13_5100140	4.56E-10	2.9	50.37–94.89	*PANK3*
	ACL	5	S13_8121375	2.55E-06	0.8	50.37–84.19	*PANK3*
17	C20:1	8	S17_8518233	0.000359	1.1	81.50–98.32	
21	C20:3	3	S21_3726359	4.10E-07	2.2	22.31–39.63	*ENO1, PIK3CD*
27	C20:3	7	S27_4705736	6.14E-07	2.2	35.27–49.58	

*Chr* Chromosome, *Nsnp* The total number of significant SNPs associated with the traits, *Top SNP* The most significantly associated SNP, *P*_value The *P*-value of the top SNP, *Var* Phenotypic variance explained by the top SNPs, *Range* Region of the chromosome that significant SNPs covered

### LD analysis for Chr13

**Fig. 4:**
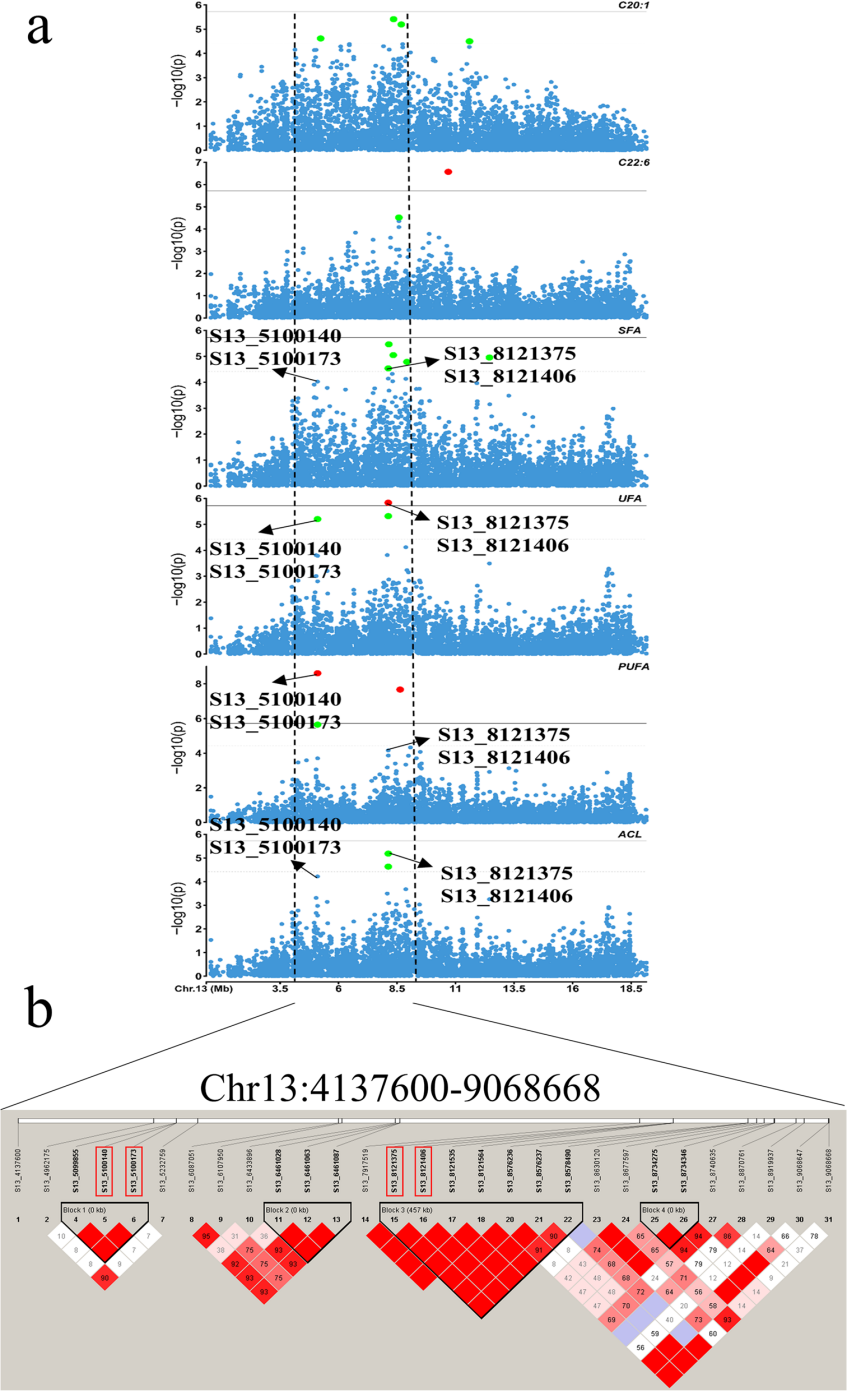
Manhattan plot for the six fatty acid composition traits located on Chr13. **A** is the Manhattan plot of the six fatty acid composition traits (C20:1, C22:6, SFA, UFA, PUFA, ACL) on Chr13. Each dot corresponds to an SNP within the data set. The horizontal solid and dashed lines represent the genome-wide significance threshold (0.05/N) and chromosome-wide suggestive threshold (1/N). The remaining SNPs with r^2^ > 0.3 and − log_10_(*P*) < 4.42 genomic regions were obtained within a total range of 4.9 Mb (Chr1:4,137,600 – 9,068,668; vertical black dashed line. **b** is LD blocks with 29 significant SNPs on Chr13 that affect the above six fatty acid composition traits. Two blocks (Chr13: 5.09–5.10 Mb and Chr13: 8.21–8.57 Mb) containing multiple traits shared SNPs (S13_8121375, S13_8121406, S13_ 5,100,140, and S13_5100173) were obtained

The above results showed that all six fatty acid composition traits (C20:1, C22:6, SFA, UFA, PUFA, ACL) were identified with significant signals within the same QTL interval (Chr13: 4.13–11.58 Mb). The four traits SFA, UFA, PUFA, and ACL shared four significantly associated SNPs (S13_8121375, S13_8121406, S13_5100140, and S13_5100173) (Fig. 4a). These four loci explained 6.2%, 6.6%, 8.4%, and 2.9% of the phenotypic variation for these four traits, respectively. Multiple traits shared the Chr 13 QTL interval, suggesting the presence of key causal mutation loci and causal genes affecting fatty acid composition traits in breast muscle within this region. Thus, we conducted further LD analysis of the Chr 13 QTL region. A total of 29 significant SNPs were obtained for the six fatty acid composition traits (C20:1, C22:6, SFA, UFA, PUFA, and ACL) and used to construct haplotypes. Two small blocks containing SNPs shared by multiple traits were obtained (Chr13:5.09–5.10 Mb and Chr13: 8.21–8.57 Mb). For the block at 5.09–5.10 Mb, there were three SNPs with high linkage with all other SNPs, and the block at 13:8.21–8.57 Mb contained seven SNPs with high linkage with all other SNPs (Fig. 4b).

### WGCNA for fatty acid traits

**Fig. 5:**
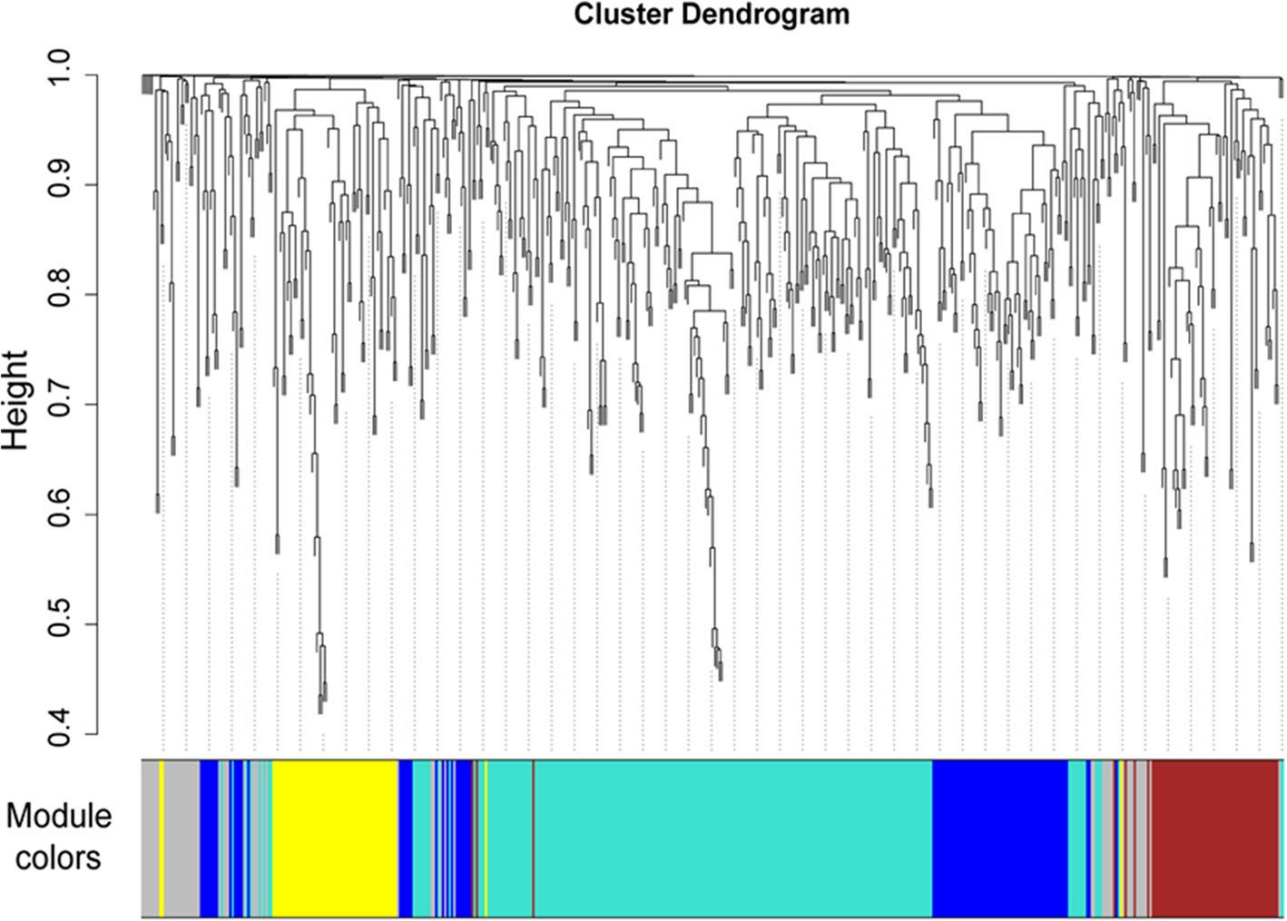
Clustering dendrogram of gene profiles from breast muscle tissues at 14, 22, and 30 weeks of age. The gene clustering dendrogram was obtained by hierarchical clustering of adjacency-based dissimilarity. Each short vertical line corresponds to a gene, and the branches are expression modules of highly interconnected groups of genes. The color row underneath the dendrogram shows the assigned original module, and each color represents a specific gene module. A total of 4 modules, ranging from 58 to 231 genes in size, were identified

**Fig. 6:**
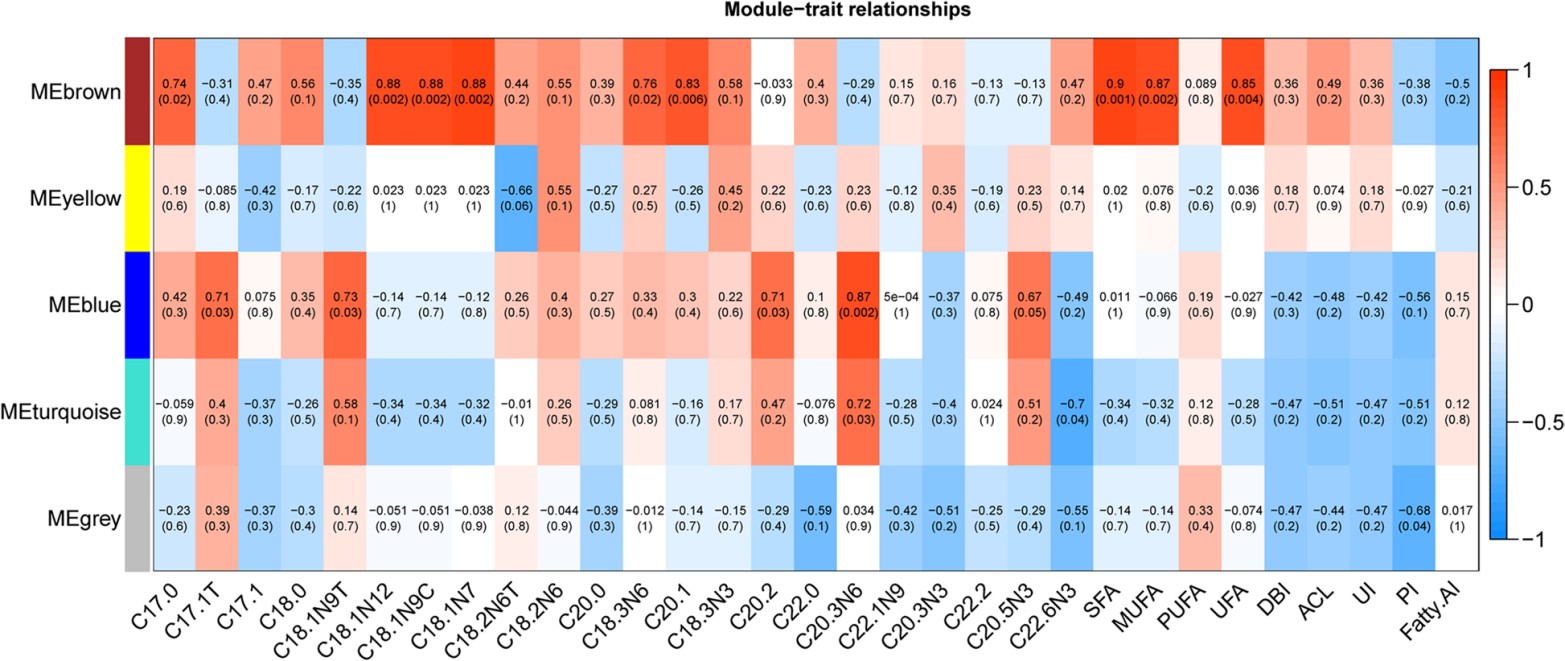
Relationships between modules and fatty acid composition traits in breast muscle of Gushi chicken. Each row in the table corresponds to a module, and each column to a trait. Each cell contains the corresponding correlation value above and the *P*-value below. The table is color-coded by correlation and the color legend. The intensity and direction of correlations are indicated on the right side of the heatmap (red, positively correlated; blue, negatively correlated)

By integrating the results of the GWAS for fatty acid traits and the transcriptome profile data of Gushi chicken breast muscle, 505 genes were identified. The expression data of these 505 genes in Gushi chicken breast muscle were used to complete the WGCNA. A total of 4 modules (turquoise, yellow, brown, and blue) were identified, and the number of genes in these modules was 231, 94, 63, and 59, respectively (Fig. 5). In addition, association analysis between these modules and fatty acid composition traits was completed. Turquoise, brown, and blue were specific transcriptional modules associated with fatty acid composition traits in breast muscle (Fig. 6). Among them, the turquoise module was significantly positively correlated with C20.3N6 (*P* < 0.05) and negatively correlated with C22:6N3 (*P* < 0.05), the blue module was significantly positively correlated with C17:1 T, C18:1N9T, C20:2 (*P* < 0.05) and highly significant positively correlated with C20:3N6 (*P* < 0.01), the brown module was significantly positively correlated with C17:0, C18:3N6 (*P* < 0.05) and highly significant positively correlated with C18:1N12, C18:1N7, C18:1N9C, C20:1, SFA, MUFA (*P* < 0.01). Unfortunately, the yellow module was not significantly correlated with any of the fatty acid composition traits.

**Fig. 7:**
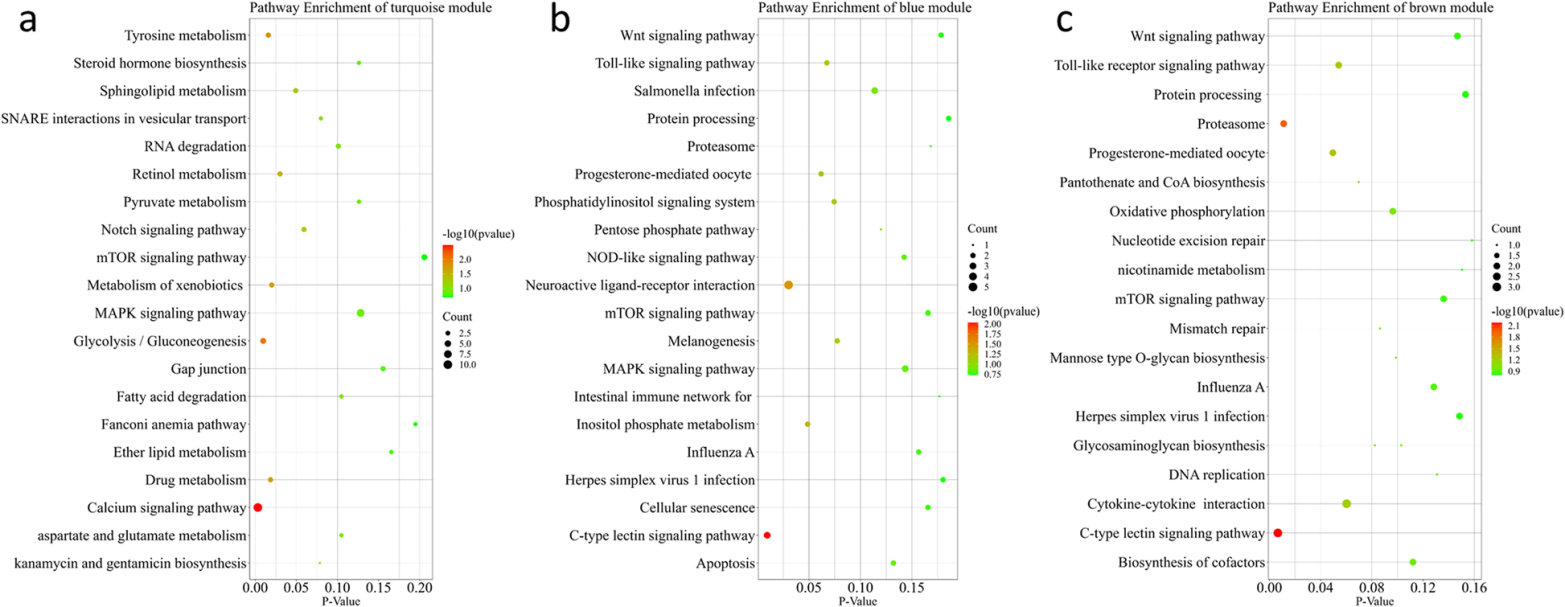
Pathway enrichment of genes in turquoise **(A)**, blue **(B)**, and brown **(C)** modules

**Fig. 8:**
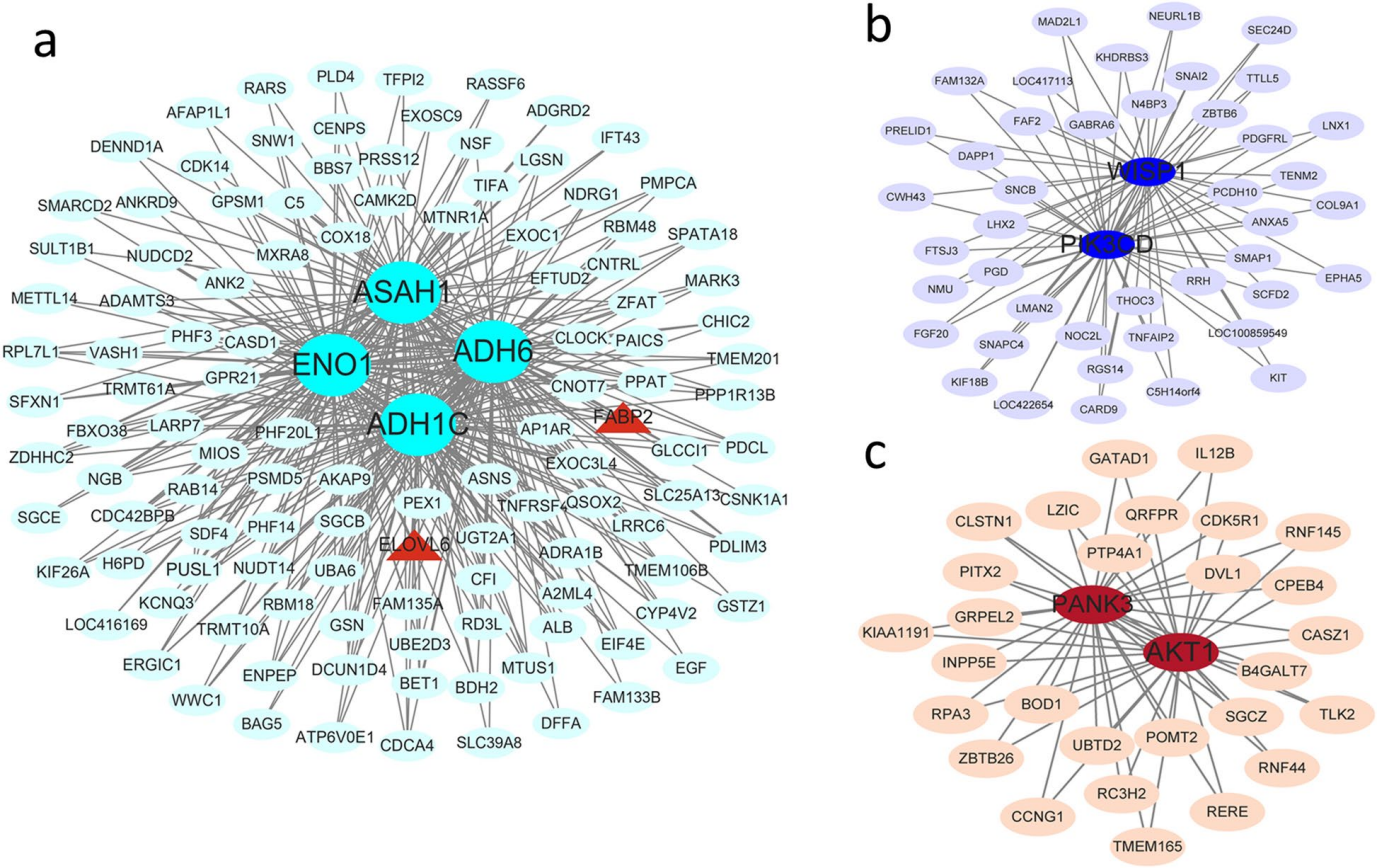
The interactive network of hub genes in the turquoise **(a)**, blue **(b)**, and brown **(c)** modules. The node size and edge number are proportional to the degree and connection strength, respectively

To elucidate the biological significance of the gene co-expression networks, GO term and KEGG pathway enrichment analysis was performed for these genes in each specific transcriptional module associated with fatty acid composition traits (Additional file 7:Table S4, Additional file 8:Table S5). KEGG pathway analysis showed that genes in these 3 modules were enriched in pathways closely related to fatty acid synthesis and decomposition. As shown in Fig. 7, genes in the turquoise module were enriched in the glycolysis/gluconeogenesis, sphingolipid metabolism, fatty acid degradation, and pyruvate metabolism pathways, and other pathways, genes in the blue module were enriched in the inositol phosphate metabolism, phosphatidylinositol signaling system, and Wnt signaling pathway, and genes in the brown module were enriched in pantothenate and CoA biosynthesis, oxidative phosphorylation, biosynthesis of cofactors and others. Combining the results of pathway analysis and the connectivity of genes in each module, we identified four hub genes in the turquoise module (*ENO1*, KME = 0.913; *ADH6*, KME = 0.819; *ASAH1*, KME = 0.807; *ADH1C*, KME = 0.800), 2 hub genes in the blue module (*PIK3CD*, KME = 0.967; *WISP1*, KME = 0.862), 2 hub genes in the brown module (*AKT1*, KME = 0.892; *PANK3*, KME = 0.867), and constructed gene co-expression network for the 3 modules (Fig. 8). These hub genes may be the causal genes regulating the synthesis and catabolism of fatty acid composition traits in chicken breast muscle.

## Discussion

Although GWAS has been widely used to explore the key genetic variations and genes of important traits in various species [16, 29, 30], the causal genes could not be identified directly and accurately. Therefore, we used the combined analysis of GWAS and WGCNA to analyze the fatty acid composition traits in Gushi chicken breast muscle and identified the key genes. It is very interesting to find in our results that the fatty acid composition traits significantly associated with the three modules were consistent with the fatty acid composition traits associated with significant SNPs in the GWAS results, which suggests that the results obtained from both analytical strategies are reliable.

A strong candidate gene *PANK3* was found in the QTL on Chr13 (4.13–11.58 Mb) shared by multiple traits, including C20:1, C22:6, SFA, UFA, PUFA, and ACL, which is also a hub gene in the brown module. Notably, brown was highly significantly correlated with SFA, UFA, and MUFA (*P* < 0.01). *PANK3* is the first step in the catalytic coenzyme A (CoA) biosynthesis pathway and is strictly regulated by feedback from acetyl CoA levels in vivo [31]. During the synthesis process, acetyl CoA is a direct feedstock for the de novo biosynthesis of fatty acids [32]. During the catabolism process, acetyl CoA is the intermediate metabolite of β-oxidative catabolism of long-chain fatty acids [33, 34]. It may slow down fatty acid synthesis in vivo by feedback inhibiting the expression of the *PANK3* gene. Elisabeth John et al. found that miR-103–1 produced by the *PANK3* was significantly induced at least fivefold during adipogenesis [35], and the *PANK3* gene was significantly (*P* < 0.05) associated with growth-development and fat deposition levels in Xinyang buffaloes by WGCNA analysis [36]. To sum up, the *PANK3* gene is closely related to fatty acid metabolism, especially SFA, UFA, and PUFA, based on our GWAS and WGCNA results.

In order to more accurately locate the causal mutation and genes affecting fatty acid composition traits in chicken muscle, we performed a haplotype analysis of the QTL on Chr13 and two important gene intervals (Chr13: 5.09—5.10 Mb and Chr13: 8.21—8.57 Mb). A strong candidate gene is located in the block, namely C1QTNF2. The C1q family was predicted to be involved in regulating the lipid metabolic process, and the deficiency of C1QTNF2 upregulates the expression of lipolytic enzyme, leading to enhanced lipolysis in adipose tissue [37–39]. Overexpression of C1QTNF2 inhibits triglyceride hydrolysis in fatty acid cells [40]. In addition, an important microRNA is located in the block, namely miR-146a. Several studies have shown that miR-146a regulates insulin sensitivity in adipocytes by down-regulate *NPR3* gene expression in mouse adipocytes and can also target *MED1* to regulate glucose and lipid metabolism in mouse hepatocytes [41, 42]. Springer CB et al. [43] showed that miR-146a expression was significantly increased (*P* = 0.02) after high-fat meals. These two blocks on Chr13 and genes within may be key regions and genes regulating the synthesis and catabolism of fatty acid composition.

Due to the different types of fatty acids that make up dietary fats, their physiological functions and health effects vary. Previous studies have suggested that total dietary fat intake was strongly associated with atherosclerosis [44], while studies on the composition of dietary fatty acids in coronary heart disease have shown that the type of dietary fat has a more significant effect on atherosclerosis than fat intake [45]. In the present study, we found significant signals for FattyAI on Chr2 (14.14–14.40 Mb) and Chr5 (49.81–52.97 Mb). We identified hub genes in these QTL regions: inducible signaling pathway protein-1 (*WISP1*) and *AKT1*. *WISP1*, located in the Wnt signaling pathway, is a newly identified adipokine [46]. It has been shown that circulating levels of WISP adipokines are higher in obese patients with increased insulin resistance and that *WIPS1* adipokines impair glucose homeostasis and induce diabetes [47]. PI3K-AKT-mTOR is a classical pathway that responds to insulin signaling and is closely linked to carbohydrate and lipid metabolism [48, 49]. *AKT1* phosphorylation activates the mammalian target of the rapamycin (mTOR) signaling pathway and subsequently enhances sterol regulatory element binding protein 1 (*SREBP1*), which increases intracellular triacylglycerol content and plays an important role in regulating the de novo synthesis of fatty acids in goat mammary epithelial cells [50]. Integrin-dependent of *AKT1* activates the expression of PGC-1α and *PDK4*, thereby enhancing fatty acid oxidation [51]. Therefore, *WIPS1* and *AKT1* may have a close relationship with human fatty acid intake on the formation of atherosclerosis, but the exact regulatory mechanism needs further study.

QTLs were found for two ultra-long chain unsaturated fatty acids (C18:1, C20:3) on Chr4, and two candidate gene intervals with 1.69 Mb (Chr4: 50.57 -52.25 MB) and 7.49 Mb (chr4: 53.48 -60.97 Mb) were defined based on 12 significant SNPs. Three hub genes (*ADH1C, ADH6*, and *ASAH1*) were identified in these two intervals. *ADH6* and *ADH1C* are both a member of the alcohol dehydrogenase family. Many studies have shown that alcohol plays a role in the oxidative decomposition and synthesis of fatty acids and is associated with the induction of many rate-limiting enzymes for fatty acid synthesis [52]. The alcohol inhibits fatty acid oxidation by inhibiting peroxisome proliferator-activated receptor and AMP-activated protein kinase[53, 54], while chronic and acute alcohol stimulation in mice increased levels of mature *SREBP*-1 protein in the liver to affected fatty acid synthesis[55, 56]. In addition, an SNP (c.-64 T > C) in *ADH1C* was sown to have an association with intramuscular fat in the longissimus thoracis muscle of cattle[57]. V. dunalianum and 6'-O-caffeoyl-arbutin were found to regulate the expression of *ADH1C* protein and play a role in lowering blood lipids in the high-fat diet-induced rat model of hyperlipidemia[58]. Thus, *ADH6* and *ADH1C* can regulate lipid synthesis and catabolism through metabolic reactions to ethanol. *ASAH1* hydrolyzes sphingolipid ceramides into sphingosine and free fatty acids at acidic pH and also catalyzes the reverse reaction allowing the synthesis of ceramides from fatty acids and sphingosine[59]. P. Lu et al. [60] showed that *ASAH1* activity is important for preventing the accumulation of long-chain ceramides such as C16-ceramide. In addition, genes known to be associated with fatty acid synthesis were identified in this region (*ELOVL6* and *FABP2*). *ELOVL6* is involved in the saturation of the monounsaturated extension of C16 and the formation of C18 [61]. A QTL locus significantly affecting linoleic acid was identified in the 58.4—58.4 Mb interval on Chr4 of Korean native chicken breast and leg muscle, which is consistent with our findings [62]. *ELOVL6* may be a potential candidate gene for the muscle C18:1n-9/C16:1n-7 and C18:1n-9/C18:0 content loci on pig chromosome 8 [63]. The FABP family is thought to play a role in the intracellular transport of long-chain fatty acids and their acyl-CoA esters [64]. Studies have shown that the *FABP2* Ala54Thr polymorphism is significantly associated with postprandial hypertriglyceridemia. Particularly in the middle-aged and elderly population, codon 54 carriers of the *FABP2* gene have a hyperlipidemic profile [65, 66]. In summary, *ELOVL6* and *FABP2* located in the QTL on Chr4 are genes known to be associated with fatty acid composition traits. *ADH6* and *ADH1C* may play a major role in fatty acid oxidation as coenzymes, indicating that genes located in the QTL on Chr4 may be the causal genes regulating the synthesis and catabolism of ultra-long chain unsaturated fatty acids.

QTLs were identified for C20:3 both on Chr21 and Chr27. Interestingly, genes within these two QTLs were located in the two modules (turquoise and blue), which also significantly correlated with C20-series fatty acids (Pearson's r2 > 0.5). The hub genes located in this gene interval in the two modules are *ENO1* and *PIK3CD*. It is known that MiR-125b can inhibit the insulin signaling pathway by targeting *PIK3CD* in hepatocytes [67]. *ENO1* encodes alpha-enolase and is involved in Glycolysis / Gluconeogenesis pathway, possibly regulating lipid synthesis through glucose restriction [68]. Thus, genes located in these two QTLs, such as *ENO1* and *PIK3CD,* may be associated with the synthesis and catabolism of C20-series fatty acids.

The Gushi-Anka F2 resource group is the second domestic line to be used for chicken genetic targeting studies. It was formed using the F-2 distant half-sib design, which is large in size and comprehensive in phenotypic determination [69]. The Gushi chicken is a high-quality meat and egg-type breed with good water-holding capacity, fine muscle fiber diameter, high muscle tenderness, high free amino acid content, tender meat, and tasty flavour [25]. Anka chickens are a fast-growing breed, with fast growth and a high percentage of UFA, especially PUFA, essential fatty acids, arachidonic acid, and linoleic acid are the characteristics of fatty acid composition in Anka chicken muscle. The two are more distantly related, with more significant variation between traits and higher heterozygosity in the F2 generation. Thus the two populations are designed for forward and backward F2 crosses, which is conducive to genetic localization studies [70]. China has abundant livestock and poultry breed resources, and it will be important to use these breeds to establish reference lines to supplement the deficiencies in genomic studies.

## Conclusion

This study first completed a GWAS analysis based on GBS sequencing data of 721 individuals in the F2 resource population. The phenotypic data of 30 fatty acid composition traits in breast muscle and 128 suggestive significantly associated SNPs for 11 fatty acid composition traits were identified, which mapped on Chr2, 3, 4, 5, 13, 17, 21, and 27. And then completed a WGCNA analysis based on the transcriptome profile of 505 genes identified in the above SNPs linkage disequilibrium region and the fatty acid composition data in breast muscle of Gushi chicken. Three specific transcription modules and eight key regulatory genes, including *ENO1*, *ADH6*, *ASAH1*, *ADH1C*, *PIK3CD*, *WISP1*, *AKT1*, and *PANK3*, related to the fatty acid composition in chicken breast muscle were identified. These results revealed the genetic structure and molecular regulatory network of fatty acid composition traits in the breast muscle of Gushi chicken and provided a basis for further elucidating the genetic regulatory mechanism and identifying molecular markers of breeding value.

## Materials and methods

### Gushi-Anka F2 resource population and phenotypic data of the fatty acid composition

The F2 resource population used in GWAS was established by Henan Poultry Germplasm Resources Innovation Engineering Research Center for genome research and consisted of four cross-bred families (Anka-cocks mated with Gushihens) and three reciprocal families (Gushi-cocks mated with Anka-hens) [71], which eventually produced a total of 860 F2 chickens and obtained phenotypic values for 21 fatty acid contents by gas chromatography. We further calculated nine fatty acid metabolic indices using the following equations [72, 73].

1 $$\mathrm{Double\,bond\,index }(\mathrm{DBI}) = \sum (\mathrm{Percentage\,of\,fatty\,acids }\times \mathrm{ Number\,of\,double\,bond})$$

2 $$\mathrm{Average\,chain\,length }(\mathrm{ACL}) = \sum (\mathrm{Percentage\,of\,fatty\,acids }\times \mathrm{ Carbon\,length})$$

3 $$\mathrm{Unsaturated\,index }(\mathrm{UI}) = \sum (\mathrm{Percentage\,of\,fatty\,acids }\times \mathrm{ Number\,of\,double\,bond }\times 2+\mathrm{ Percentage\,of\,fatty\,acids }\times \mathrm{ Number\,of\,triple\,bond }\times 4 + \dots )$$

4 $$\begin{array}{c}\mathrm{Peroxide\,index }(\mathrm{PI}) = (\mathrm{\%Monoenoic }\times 0.025)+ (\mathrm{\%Dienoic }\times 1)\\ + (\mathrm{Trienoic }\times 2) + (\mathrm{\%Tetraenoic }\times 4 )\\ + (\mathrm{\% Pentaenoic }\times 6) + (\mathrm{\%Hexaenoic }\times 8)\end{array}$$

5 $$\mathrm{Fatty\,acid\,atherogenic\,index }(\mathrm{FattyAI}) = (4 \times \mathrm{ C}14:0 +\mathrm{ C}16:0) / (\mathrm{MUFA }+\mathrm{PUFA})$$

where SFA is saturated fatty acids that including laureate (C12:0), myristic acid (C14:0), pentadecanoate (C15:0), palmitate (C16:0), heptadecanoate (C17:0), stearate (C18:0), arachidonate (C20:0), behenate (C22:0); MUFA is monounsaturated fatty acids that including Palmitoleate (C14:1), Oleate (C18:1), Eicosenoate (C20:1), Erucic acid (C22:1); and PUFA is polyunsaturated fatty acids that including palmitic acid (C16:2), Linolelaidic acid (C18:2), Gamma Linolenate (C18:3), 11–14 Eicosadienoate (C20:2), 11–14-17 Eicosatrienoate (C20:3), Arachidonate (C20:4), cis-13,16, 19-Docosatrienoic acid (C22:3), Docosatetraenoic acid (C22:4), Docosahexaenoate (C22:6); UFA is the sum of MUFA and PUFA. Before the association analysis, these 9 fatty acid metabolic traits were log2-transformed and then transformed data were used in the following genetic analyses.

### Transcriptome profile and fatty acid composition in breast muscle of Gushi chicken

WGCNA was performed using transcriptome profiles and fatty acid composition data of Gushi chicken breast muscle. We completed the transcriptome sequencing in 14, 22, and 30 weeks of age and identified a total of 16,755 known genes previously [21]. The total number of samples taken into the study for transcriptome analysis is 9. The same breast muscle samples as the transcriptome sequencing, with three biological replicates at each developmental stage, were sent to Suzhou PANOMIX Biomedical Tech Co., LTD and performed the fatty acid targeting analysis by Trace 1310-ISQ 7IQS gas-mass spectrometer (Thermo, USA) to obtain the dynamic change pattern of fatty acid composition in breast muscle of Gushi chickens at 14, 22, and 30 weeks of age (Additional file 4: Table S1). The gas chromatographic conditions used were an injection volume of 1μL, injection temperatures of 250℃, ion source temperature of 230℃, transmission line temperature of 250℃, and quadrupole temperature of 150℃. The programmed temperature rise starts at 80 °C and is maintained for 1 min, followed by a 20 °C/min rise to 160 °C for 1.5 min, 3 °C/min rise to 196 °C for 8.5 min, and a final 20 °C/min rise to 250 °C for 3 min. The carrier gas was helium at a flow rate of 0.63 mL/min.

### Genotyping, imputation and quality control

Genomic DNA was extracted from blood samples by the Qiagen DNeasy Blood and Tissue Kit (Qiagen, Hilden, Germany) according to the manufacturer's instructions. All DNA samples were digested with a combination of EcoRI and MseI for double-digest [74]. A total of 768 chickens from the F2 resource population were genotyped using the Illumina HiSeq X Ten platform (PE150) according to the manufacturer’s protocol. The quality control (QC) procedures were carried out using VCFtools (version 0.1.13) [75], SNPs were identified using the TASSEL GBS analysis pipeline (version 5.2.31) [76, 77], and SNPs with call rates > 0.30 and minor allele frequencies > 0.05, genotypes with a quality above 98 (minGQ ≥ 98) and depth ≥ 5, consistent with Hardy–Weinberg equilibrium; and max missing rate < 0.40 samples with genotyping were retained for further statistical analyses. Filtered paired reads were aligned to the chicken reference genome Gallus_gallus-6.0(released in 2018) using Bowtie2 (version 2.3.0) [78]. A total of 7,258 million clean reads and 6,071 million good barcode reads were obtained after 768 samples were sequenced. The genome coverage at least 1X was 58.462% by the GBS. After strict parameter filtering in the TASSEL-BEAGLE-GBS pipeline (including imputation) and removal of the sex chromosomes, we identified 323,306 SNPs ultimately (average sequencing depth was 13 ×). The average SNP density and average SNP variant rate per chromosome were 309 SNPs/Mb and 5.79 kb/SNP, respectively (Additional file 3:Fig. S3).

### Single trait GWAS

Population structure is the main origin of confounding effects in genetic analyses. Principal component analysis (PCA) was performed using genome-wide complex trait analysis (GCTA) [79] software to assess population structure, and the first two principal components (PCs) were then calculated and used as covariates in the mixed model. The variances explained by PC1 and PC2 were 18.7% and 11.3%, respectively. A genomic relationship matrix was constructed with the 323,306 SNPs using GCTA software and used as random effects in the mixed model.

GWAS analysis of 30 fatty acid composition traits was carried out in the GCTA program using a mixed linear model (MLM).

The following MLM were used:

$${\varvec{y}}=\boldsymbol{ }{\varvec{W}}\boldsymbol{\alpha }\boldsymbol{ }+\boldsymbol{ }{\varvec{\beta}}{\varvec{x}}\boldsymbol{ }+\boldsymbol{ }{\varvec{u}}\boldsymbol{ }+\boldsymbol{ }{\varvec{e}}$$

where y is the phenotypic value of each trait; W is a matrix of covariates (fixed effects) controlling for population structure (first two PCs), sex, and batch effects; α is a vector containing the corresponding coefficients of the intercept; β is the SNP effect and x is the carrier of the SNP genotype; u is a vector of random effects with a covariance structure, obeying a normal distribution as u ~ N (0, KVg), where K is known of the genetic relationship matrix; e is a random error vector.

The genome-wide significance thresholds were calculated using a valid number of independent SNPs using the Bonferroni correction. Genome-wide independent markers were calculated using PLINK [80] -indep-pairwise with a window size of 25 SNPs, a step size of 5 SNPs, and an r^2^ threshold of 0.1. The genome-wide significance threshold is 1.90E-06 (0.05/26,430; -log_10_(*P*) > 5.72) and the chromosome-wide suggestive threshold is 3.78E-05 (1/26,430; -log_10_(*P*) > 4.42), based on 26,430 independent SNPs markers. Manhattan and Q-Q plots were drawn from GWAS results using the CMplot package (https://github.com/YinLiLin/R-CMplot) within the R software (http://www.r-project.org/).

### WGCNA for fatty acid composition traits

Genes located in the linkage disequilibrium genomic region of the 128 suggestive significantly associated SNPs identified by GWAS were extracted and combined with the breast muscle transcriptome data for joint analysis. Finally, 505 genes were obtained for WGCNA. Gene co-expression networks were constructed by the WGCNA package within the R software [81]. Firstly, the Pearson correlation coefficients of each gene pair were calculated to build a Pearson correlation coefficient matrix, and then the key parameter β values were optimized up to the plateau (β = 16) to construct a weighted neighborhood matrix. The expression correlation values were used to convert the adjacency Matrix into a topological overlap measure (TOM), and hierarchical clustering was performed based on the TOM. Ultimately, a dynamic tree-cutting algorithm was used to identify modules with a minimum number of genes of 32. Subsequently, Gene ontology (GO) terms and Kyoto Encyclopedia of Genes and Genomes (KEGG) pathways analyses of genes in the module were performed by the Clusterprofiler package with a corrected *P*-value < 0.05 and were considered to be significantly enriched [82–85].

To integrate the two strategies of GWAS and WGCNA to identify the key regulatory genes of fatty acid composition in Gushi chicken breast muscle, we selected 22 fatty acids with the same carbon chain length from the data of Gushi chicken breast muscle fatty acid composition targeting analysis based on the carbon chain length of fatty acids associated with significant SNPs in GWAS, including C17:0, C17:1 T, C17:1, C18:0, C18:1N9T, C18:1N12, C18:1N9C, C18:1N7, C18:2N6T, C18:2N6, C20:0, C18:3N6, C20:1, C18:3N3, C20:2, C22:0, C20:3N6, C22 C20:1N9, C20:3N3, C22:2, C20:5N3, C22:6N3. The contents of the above 22 fatty acids and 9 fatty acid metabolism indicators, including SFA, MUFA, PUFA, UFA, DBI, ACL, UI, PI, and FattyAI, in breast muscle of Gushi chicken at 14, 22, and 30 weeks of age were used as the phenotypic data, for the correlation analysis with gene co-expression module. Pearson correlation coefficients were used to evaluate the correlation between each module and each fatty acid composition trait. *P* < 0.05 was set as the threshold standard for a significant correlation between modules and traits. Hub genes were identified based on the following principles: (1) the eigengene connectivity (KME) value > 0.8; (2) TOM value > 0.2; (3) the genes obtained from the above two principles and whose functional annotations associated with fatty acid composition traits. The gene regulatory network in the key module was drawn by using Cytoscape software [86].

### Supplementary Information

**Additional file 1. **

**Additional file 2. **

**Additional file 3. **

**Additional file 4. **

**Additional file 5. **

**Additional file 6. **

**Additional file 7. **

**Additional file 8. **

## Data Availability

All raw sequence data have been deposited in the NCBI Sequence Read Archive database with the accession number SRR12532401–SRR12532408 (BioProject number PRJNA659316). Data generated or analysed during this study are included in this published article and its supplementary information files. We declare that the data supporting the findings of this study are available within the article and its supplementary information files.

## Abbreviations

GWAS: Genome-wide association study
WGCNA: Weighted gene co-expression network analysis
SNPs: Single nucleotide polymorphisms
GBS: Genotyping by sequencing
QTL: Quantitative trait loci
SFA: Saturated fatty acid
UFA: Unsaturated fatty acids
MUFA: Monounsaturated fatty acids
PUFA: Polyunsaturated fatty acids
DBI: Double bond index
ACL: Average chain length
UI: Unsaturated index
PI: Peroxide index
FattyAI: Fatty acid atherogenic index
LD: Linkage disequilibrium
RNA-Seq: RNA sequencing
eQTL: Expression quantitative trait loci
QTTs: Quantitative trait transcripts
QC: Quality control
GCTA: Genome-wide complex trait analysis
PCs: Principal components
MLM: Mixed linear model
TOM: Topological overlap measure
GO: Gene ontology
KEGG: Kyoto Encyclopedia of Genes and Genomes
KME: The eigengene connectivity
SIFT: Sorting Intolerant From Tolerant
CoA: Coenzyme A
SREBP1: Sterol regulatory element binding protein 1
IMF: Intramuscular fat
