# Supplementary material for: Genetic architecture and key regulatory genes of fatty acid composition in Gushi chicken breast muscle determined by GWAS and WGCNA
Source: BMC Genomics. 2023 Aug 3;24:434. doi: 10.1186/s12864-023-09503-1 (PMC10398928; doi:10.1186/s12864-023-09503-1)
Supplement: Supplementary file 2 — Additional file 2. [file 12864_2023_9503_MOESM2_ESM.doc]

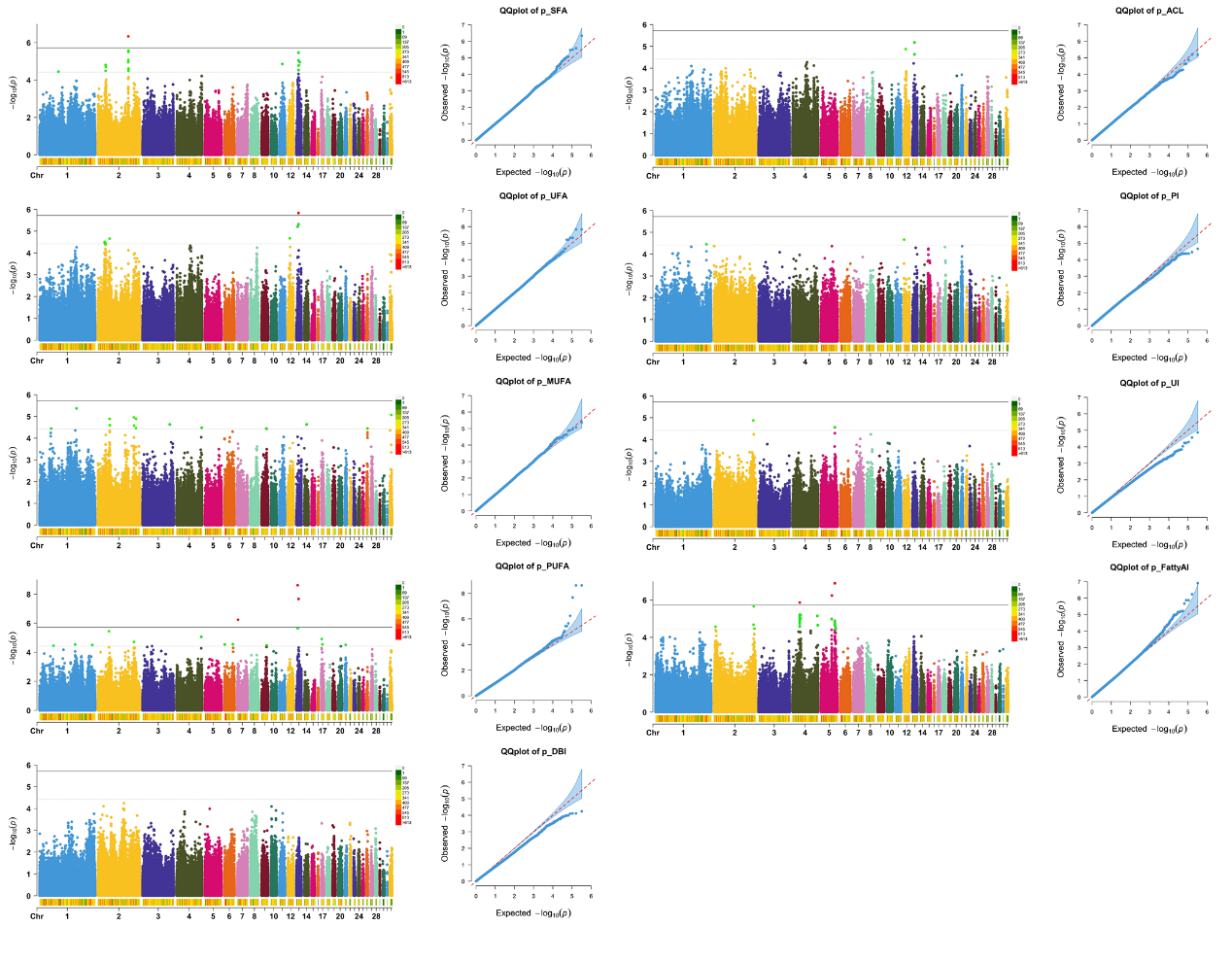


**Additional file 2:Fig. S2. The Manhattan and Q-Q plots for 9 fatty acid metabolic traits.** Each dot in this figure corresponds to an SNP within the data set. In each Manhattan plot, the dot color indicates the chromosome on which the SNP is located, the dot position indicates the -log_10_-transformed *P* value of the SNP, the number below represents the chromosome number, the length of the figure above the number represents the length of the chromosome, and the color represents the number of SNPs on the chromosome. The solid and dashed lines represent genome-wide significance (-log_10_(*P*) > 5.72) threshold and chromosome-wide suggestive threshold (-log_10_(P) > 4.42), respectively. For each Q-Q plot, the *x-axis* represents the expected -log_10_-transformed *P* value, the *y-axis* shows the observed -log_10_-transformed *P* value, and the red line is the diagonal line.
