# Supplementary material for: Genetic architecture and key regulatory genes of fatty acid composition in Gushi chicken breast muscle determined by GWAS and WGCNA
Source: BMC Genomics. 2023 Aug 3;24:434. doi: 10.1186/s12864-023-09503-1 (PMC10398928; doi:10.1186/s12864-023-09503-1)
Supplement: Supplementary file 3 — Additional file 3. [file 12864_2023_9503_MOESM3_ESM.doc]

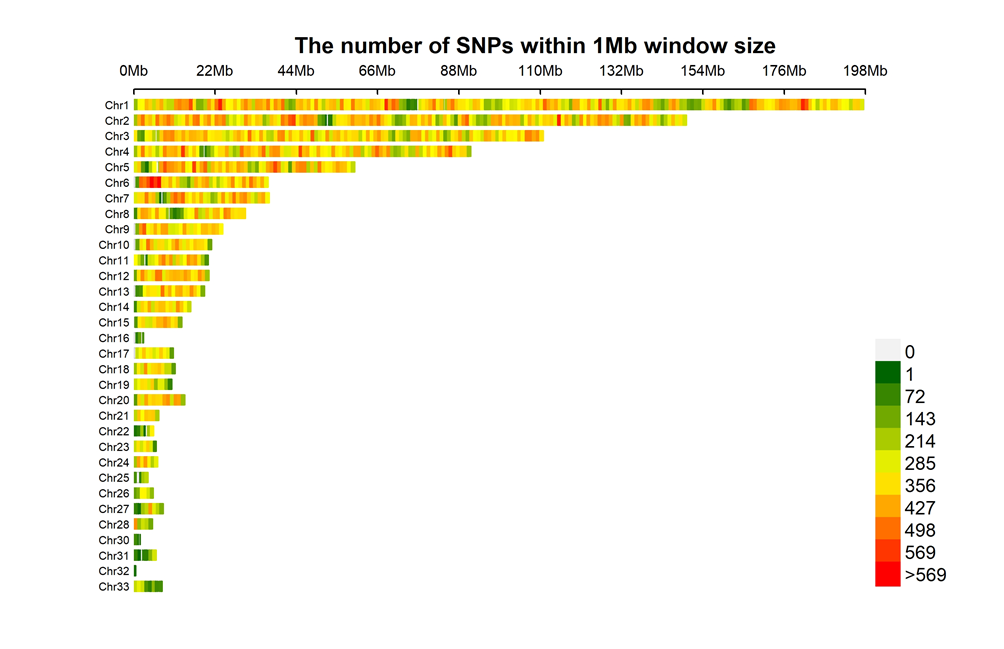


**Additional file 3:Fig. S2. Density of SNPs in the chicken genome.** SNP density plot chromosome wise representing number of SNPs within 1 Mb window size. The horizontal axis shows the chromosome length (Mb); the different color depicts SNP density.
